# Osteomodulin positively regulates osteogenesis through interaction with BMP2

**DOI:** 10.1038/s41419-021-03404-5

**Published:** 2021-02-01

**Authors:** Wenzhen Lin, Xiaohan Zhu, Li Gao, Mengying Mao, Daming Gao, Zhengwei Huang

**Affiliations:** 1 Department of Endodontics, Shanghai Ninth People’s Hospital, College of Stomatology, Shanghai Jiao Tong University School of Medicine, Shanghai, China; 2 National Clinical Research Center for Oral Diseases, Shanghai, China; 3 Shanghai Key Laboratory of Stomatology & Shanghai Research Institute of Stomatology, Shanghai, China; 4 Department of Endodontics, Shanghai Stomatological Hospital, Fudan University, Shanghai, China; 5 Oral Biomedical Engineering Laboratory, Shanghai Stomatological Hospital, Fudan University, Shanghai, China; 6 State Key Laboratory of Cell Biology, CAS Key Laboratory of Systems Biology, CAS Center for Excellence in Molecular Cell Science, Shanghai Institute of Biochemistry and Cell Biology, Chinese Academy of Sciences, Shanghai, China; 7 University of Chinese Academy of Sciences, Beijing, China

**Keywords:** Extracellular signalling molecules, Mesenchymal stem cells

## Abstract

Osteomodulin (OMD), a member of the small leucine-rich proteoglycan family, distributes in mineralized tissues and is positively regulated by bone morphogenetic protein 2 (BMP2). However, the exact function of OMD during mineralization and its association with BMP2 remain poorly understood. Herein, the expression pattern of OMD during osteogenesis was investigated in human dental pulp stem cells. Silencing *OMD* gene significantly suppressed the alkaline phosphatase activity, mineralized nodule formation and osteogenesis-associated gene transcription. Besides, OMD could enhance BMP2-induced expression of *SP7* and *RUNX2* with concentration dependence in vitro. Rat mandibular bone defect model revealed that scaffolds injected with the combination of OMD and suboptimal BMP2 exhibited more mature and abundant mineralized bone than that treated with OMD or suboptimal BMP2 alone. Mechanistically, OMD could bind to BMP2 via its terminal leucine-rich repeats and formed complexes with BMP2 and its membrane receptors, thus promoting BMP/SMAD signal transduction. In addition, OMD was a putative target gene of SMAD4, which plays a pivotal role in this pathway. Collectively, these data elucidate that OMD may act as a positive coordinator in osteogenesis through BMP2/SMADs signaling.

## Introduction

Oral and maxillofacial bone defects caused by congenital disease, cancer, trauma, infection and other reasons seriously affect the quality of life^1,2^. Tissue engineering has introduced new hopes as combination of stem cells, scaffolds, and growth factors for functional bone regeneration^3^, among which growth factors are crucial in promoting bone healing^4^. However, translation of growth factors into clinical treatments has been hindered by their rapid release kinetics, poor protein stability or potential adverse effects with respect to high dose required^5^. For instance, BMP2, a leading bone graft substitute, has been associated with an increasing side effect profile because of supraphysiologic dosage^6^. In osseous tissue, the local presentation and spatiotemporal distribution of growth factors are finely orchestrated by extracellular matrix (ECM), which functions as a ligand “reservoir” by binding numerous growth factors^7^. ECM components modulate the bioactivities of growth factors and cytokines thereby regulating the nature, intensity, and duration of signaling cascades within cells^8^, and are likely to be promising in bone bioengineering field.

Small leucine-rich proteoglycans (SLRPs) are biologically active constituents of the ECM. The core proteins of the SLRPs consist of leucine-rich repeats (LRRs) flanked by two cysteine-rich clusters^9^. The pericellular localization of SLRPs, along with their multivalent binding abilities, allow for cell–matrix interactions by directly interfering with cell surface receptors and matrix molecules such as cytokines, chemokines and growth factors, leading to modulation of cellular functions^10^. SLRPs take specific roles designated during all phases of bone formation, including cellular growth, organic matrix assembly, mineral deposition, and remodeling^11^. Biglycan, for instance, has been confirmed as a modulator of the Wnt signaling, transforming growth factor (TGF)-beta and BMP pathway by binding multiple molecules^12,13^. Nevertheless, the biological mechanisms of other members in SLRPs family have not yet been fully deciphered.

Osteomodulin (OMD) (also termed osteoadherin, OSAD) is a class II keratan sulfate SLRP expressed in mineralized tissues, including bones and teeth^14,15^. Besides the hydroxyapatite binding capacity, a favorable function of OMD in governing the shape of collagen fibrils has been observed^16,17^. OMD also takes a part in the biological processes including cell adhesion^15^ and tooth formation^18^, and is involved in bone diseases such as osteoarthritis and heterotopic ossification^19–21^. Additionally, OMD has been reported to be upregulated by BMP2, a potent osteoinductive cytokine, and serve roles in the apoptosis and growth of osteoblast cells^22,23^. Our previous work has highlighted the necessity of OMD in osteo/odontoblastic differentiation of human dental pulp stem cells (hDPSCs)^24^. However, our understanding of the precise mechanism through which OMD regulates osteogenesis remains limited.

Based on the observations that OMD and TGF-beta/BMP signaling are both associated with skeletal and dental tissues, here we propose that the proteoglycan may play a direct role in modulating TGF-beta/BMP pathway. It is hypothesized that OMD is also capable of cell–matrix interactions similar to other SLRPs. In the present work, the function and expression pattern of OMD during cytodifferentiation further strengthens its role as a mineralization specific marker. The exact relationship between OMD and BMP/BMPR/SMADs pathway was further explored. We identified that OMD binds to BMP2 via the tenth and eleventh LRRs and also forms complexes with BMP receptors thereby activating downstream SMADs to regulate transcriptional response. The formation of new bone was augmented by scaffolds injected with a combination of OMD and suboptimal BMP2 in a rat mandibular bone defect model. These data demonstrate for the first time that OMD may function as a coordinator of BMP2 in osteogenesis and provide fresh perspectives in the biologic role and therapeutic potential for SLRPs.

## Materials and methods

### Cell cultu**r**es

This study was approved by the Ethics Committee of Shanghai Ninth People’s Hospital affiliated with Shanghai Jiao Tong University, School of Medicine, China (Document No. 201769). The healthy and intact third molars were obtained from individuals at the age of 18–22 for prophylactic purpose at the oral surgery clinic of the Ninth People’s Hospital affiliated to Shanghai Jiao Tong University School of Medicine. Written informed consent was obtained from each volunteer. Cell isolation and culture were performed and the multilineage differentiation ability of hDPSCs was confirmed as described previously^24^. Briefly, cells were cultured in growth medium (GM): high-glucose Dulbecco’s modified Eagle’s medium (Gibco-BRL, Gaithersburg, MD, USA) supplemented with 10% fetal bovine serum (Gibco-BRL), 100 U/mL penicillin, and 100 mg/mL streptomycin. For osteogenesis, hDPSCs were subcultured in human mesenchymal stem cell osteogenic differentiation medium (OM) (Cyagen Biosciences, Guangzhou, Guangdong, China) for up to 21 days.

### **O**MD ELISA assay

Cells were seeded in 6-well plates (2 × 10^5^ cells per well) and cultured in GM or OM for 1, 3, 7, 14 or 21 days. Supernatants were harvested 24 h after culture mediums were refreshed, and stored at −80 °C. OMD concentration was measured by Human Osteomodulin ELISA Kit (OmnimAbs, Alhambra, CA, USA) according to the kit instructions. The optical density at 450 nm was read using a microplate reader (Bio-Tek, Winooski, VT, USA) within 15 min.

### **A**lkaline phosphatase (ALP) staining, activity measurement, and Alizarin Red S (ARS) staining

hDPSCs were seeded in 24-well plates (2 × 10^4^ cells per well) and cultured in OM for 7 days. Then, cells were washed with phosphate-buffered saline (PBS) twice and fixed by 4% paraformaldehyde (PFA) fix solution (Sangon Biotech, Shanghai, China) for 30 min. The ALP staining was carried out according to the manufacturer’s instructions (Beyotime, Shanghai, China). The ALP activity was also measured using Alkaline Phosphatase Assay Kit (Beyotime). The ARS staining was performed after cells were cultured in OM for up to 21 days. Then, cells were washed with PBS and fixed by 4% PFA for 30 min. 0.5% ARS solution was added to visualize calcium deposition. Cells were incubated for 15 min at room temperature and washed to stop the reaction. Images were taken under the microscope (Leica Microsystems, Weztlar, Hessen, Germany).

### **C**ell viability assay

The cytotoxic effects of OMD were determined using a Cell Counting Kit-8 (CCK8) (Dojindo Lab, Kumamoto, Japan) according to the manufacturer’s protocol. hDPSCs were plated in 96-well plates at a density of 4 × 10^3^ cells per well. Cells were then treated with different concentrations of OMD (0, 0.385, 0.75, 1.5, 3, 6, 12 μg/mL) for 24 h or 48 h. Ten microliters CCK8 buffer was added to each well, and cells were incubated at 37 °C for 2 h. The absorbance was then measured at a wavelength of 450 nm using a microplate reader (Bio-Tek).

### **Q**uantitative real-time polymerase chain reaction (qPCR)

Total RNA of hDPSCs was extracted using TRIzol reagent (Invitrogen, Carlsbad, CA, USA) according to the manufacturer’s instruction. Isolated RNA was quantified on a Nanodrop spectrophotometer (Thermo Scientific, Wilmington, NC, USA). In all, 500 ng RNA with 260/280 ratio of ~2.0 was reverse transcribed into complementary DNA (cDNA) using PrimeScript RT reagent Kit (Takara, Kusatsu, Japan). The gene transcription level was determined using TB Green Premix Ex Taq (Takara) on a LightCycler 480 II instrument (Roche, Basel, Switzerland). Reaction program was set at 40 cycles of denaturation at 95 °C for 5 s, annealing at 55 °C for 30 s, and extension at 72 °C for 30 s. Each reaction was performed in triplicate. Melting curve analysis and agarose gel electrophoresis were performed to verify the amplified products. The expression level of mRNA was quantified and normalized to β-actin using the 2^−ΔΔCt^ method. The sequences and resources of the primers are listed in Supplemental Table 1.

### **P**lasmids and chemical reagents

cDNAs of human OMD, BMPRIA, BMPRIB, BMPRII were generous gifts from Dr. Jiahuai Han (Xiamen University, Xiamen, China). BMP2 cDNA was kindly provided by Dr. Weiguo Zou (University of Chinese Academy of Sciences, Shanghai, China). The cDNAs were inserted into pLEX-HA, pLEX-FLAG or pCMV-FLAG vectors. Five OMD deletion constructs (Δ1–5), which deleted specific LRRs, were generated by a two-step PCR method, inserted into pLEX-FLAG vectors and verified by DNA sequencing. Sequences for constructing small-hairpin RNA (shRNA) targeting human OMD were obtained from the RNAi Consortium of the Broad Institute (http://www.broadinstitute.org/rnai/trc), and shRNA plasmid was generated with pLKO.1 vector as described previously^24^.

### RNA interference and lentiviral production

The silence of *OMD* gene was produced by small-interfering RNAs (siRNAs) and shRNA. hDPSCs were transfected with siRNA using X-tremeGENE siRNA Transfection Reagent (Roche) at 50 nM final concentration according to the manufacturer’s protocol. The siRNA sequences are shown in Supplemental Table 2 and knockdown efficiency is indicated in Supplemental Fig. 1. To generate stable cell lines, lentiviruses were produced to overexpress or silence *OMD* gene using a three-plasmid packing system. The shRNA targeting *OMD* gene was constructed and knockdown efficiency was confirmed as previously described^24^. Lentiviral vector-mediated overexpression of OMD was validated by immunoblotting (Supplemental Fig. 2). Briefly, full-length OMD cDNA in pLEX plasmid was co-transfected into 293T cells together with psPAX2 packaging and pMD2. G envelope plasmid DNA. Lentivirus was harvested at 48 h after transfection and were cleansed by 0.45 μm filter. 10 μg/mL polybrene (Sigma, Saint Louis, Missouri, USA) was added into lentivirus solution before infecting cells, and stable cell lines were selected out in 1 μg/mL puromycin (Selleck chemicals, Houston, TX, USA) for 48 h.

### **I**mmunoblots (IB) and Immunoprecipitation (IP)

Cells were harvested with EBC lysis buffer (50 mM Tris HCl, pH 8.0, 120 mM NaCl, 0.5% Nonidet P-40) supplemented with protease and phosphatase inhibitors (Selleck chemicals). The protein concentration in the supernatant was determined by Bradford protein assay. 30 μg of whole-cell lysates (WCL) were separated by sodium dodecyl sulfate polyacrylamide gel electrophoresis gel, transferred onto a polyvinylidene fluoride membrane and blotted with primary antibodies. MG132 (Selleck chemicals) was added 12 h prior to cell harvest for IP analysis. Aliquots containing 500 μg proteins of each sample were incubated with anti-FLAG M2-agarose beads (Sigma) or anti-HA agarose beads (AOGMA, USA) for 3 h at 4 °C on a rotator. After centrifugation, IP complexes were washed four times with NETN buffer (20 mM Tris, pH 8.0, 100 mM NaCl, and 0.5% NP-40, 1 mM EDTA) and solubilized in loading buffer. The details about primary antibodies are listed in Supplemental Table 3.

### **C**hromatin immunoprecipitation (ChIP) assay

ChIP was performed using ChIP-IT high sensitivity kit (Active Motif, Carlsbad, CA, USA). In all, 4 × 10^6^ hDPSCs without stimulation were processed following the manufacturer’s protocol. Chromatin was sheared to a size of 200–1000 bp using a VCX-130 sonicator (Sonics and Materials, Newtown, CT, USA). Shearing Conditions were set at 25% amplitude and pulse for 2 s on and 5 s off for a total sonication “on” time of 10 min. ChIP-IT qPCR analysis kit (Active Motif) was used to determine the enrichment ratio of binding. The negative control (NC) primers in the kit served as an internal control for the ChIP reaction. Regions of the human *OMD* promoter that contained putative SMAD4 binding site predicted by FIMO program (http://meme-suite.org/doc/fimo.html) were amplified using specific primers listed in supplemental Table S1. Amplification data was quantified using DNA standard curve method according to the manufacturer’s instructions. Afterward, the products were verified by 2% agarose gel electrophoresis.

### **A**nimals

Six-week-old Sprague-Dawley (SD) male rats weighing approximately 200 g were obtained from Shanghai Experimental Animal Center. Each group consisted of five rats. Rats were fed with normal chow and water ad libitum in SPF environment. All animal protocols were approved by the Animal Experimental Ethics Committee of the Ninth People’s Hospital affiliated with the Shanghai Jiao Tong University School of Medicine (Shanghai, China) (Document No. SH9H-2019-A729-1) and implemented in accordance with the ethical standards laid down in the 1964 Declaration of Helsinki and its subsequent amendments.

### **A**nimal surgery

To fully confirm the osteogenesis potential of OMD, a full-thickness mandible bone defect model was used in SD rats. Puramatrix hydrogel (BD Biosciences, Franklin Lakes, NJ, USA) was used as the scaffold. In all, 0.1 μg of BMP2, closing to physiologic concentration, is regarded as “suboptimal” dose in rat mandibular bone defect model for limited osteogenesis exhibition^25^. The SD rats were randomly divided into five group: Sham operation group; Puramatrix (Control) group; Puramatrix + 0.1 μg BMP2 group; Puramatrix + 5 μg OMD group; Puramatrix + 0.1 μg BMP2 + 5 μg OMD group. Recombinant proteins are commercially available (Supplemental Table 4). All efforts were made to minimize the animals’ suffering. A full-thickness mandible bone defect of 3 mm in diameter was created with a dental trephine when animal was anesthetized (Fig. 2B). The defects were then filled with scaffolds containing the proteins as described before^26^. The SD rats were sacrificed at 8-week post surgery. The right mandibles were harvested and fixed in 4% PFA for 24–48 h and then stored in 70% ethanol at 4 °C for further analysis. Investigators were blinded to the groups at time of euthanasia.

### **M**icro-computed tomography (Micro-CT) evaluation

To observe new bone formation, the collected right mandibles were observed by micro-CT (Skyscan1076, Kontich, Belgium). All of the samples were scanned using 18 μm^3^ isotropic voxel size, 40 kVp peak X-ray tube potential and 240 ms integration time, and were subjected to Gaussian filtration. The quantitative measurements such as bone volume per tissue volume (BV/TV), bone mean density (BMD), trabecular thickness (Tb.Th), trabecular number (Tb.N), and trabecular separation (Tb.Sp) were calculated at the mandible bone in a region of 3 mm in diameter. A threshold of 90 was used to manually delineate bone from surrounding soft tissue. Three-dimensional (3D) images were reconstructed by NRecon Version: 1.7.1.0. The investigators were blinded to treatment of subjects.

### **H**istology and immunohistochemistry study

The rat mandibles were decalcified in 15% EDTA–2Na solution (PH 7.4) on an orbital shaker at 37 °C for 4 weeks. Tissues were embedded in paraffin and sliced into 4 μm thick sections parallel to the buccal surface of the bone. The tissue sections were deparaffinized and stained with hematoxylin and eosin (H&E) and Masson’s trichrome stain kit (Servicebio, Wuhan, Hubei, China) to detect newly formed bone and osteoids. For immunohistochemistry, tissue sections were stained following the standard protocol using anti-Osteocalcin (OCN) antibody (Santa Cruz Biotechnology, Santa Cruz, CA, USA). The Dako REAL^TM^ EnVision^TM^ Detection System (Dako, Glostrup, Denmark) was used to visualize the secondary antibody.

### **S**tatistics

Data were analyzed with the use of GraphPad Prism 8.0.2 software (GraphPad, La Jolla, CA, USA). Data were presented as mean ± SD. Differences between two groups were compared by Student’s *t*-test. When more than two groups were compared, ordinary one-way ANOVA Tukey’s multiple comparison test was applied. Data sets from in vivo analysis were based on groups of animals. Micro-CT was analyzed using Brown-Forsythe or Welch’s ANOVA test with Games-Howell’s test for multiple comparisons after normality and homogeneity of variance test. For all statistical tests, a two-sided *p*-value of < 0.05 was considered to be statistically significant.

## Results

### **O**MD promotes osteogenic differentiation and mineralization in hDPSCs

**Fig. 1:**
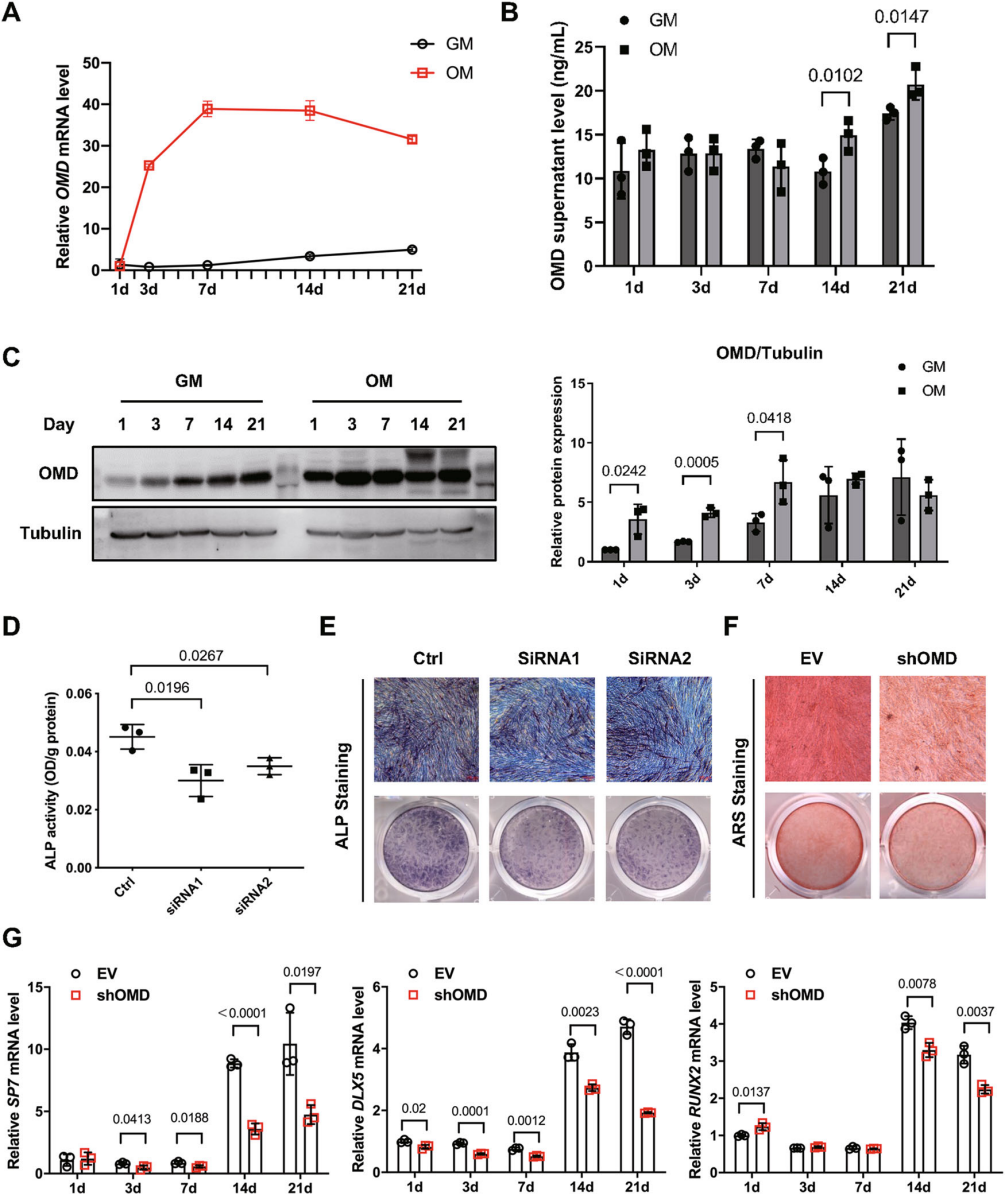
OMD is essential for the osteogenic differentiation of hDPSCs. **A** The transcriptional level of *OMD* in hDPSCs after cultured in growth medium (GM) or osteogenic medium (OM) for 1, 3, 7, 14, 21 days, respectively. **B** The concentrations of OMD in culture supernatants collected at five time points. **C** The variation of OMD protein levels in cells cultured in GM or OM and normalized to Tubulin expression by densitometric analysis. ALP activity (**D**) and ALP staining (**E**) of hDPSCs after siRNA transfection targeting *OMD* and culture with OM for 7 days (200X). **F** ARS was performed on day 21 of induction after *OMD* was silenced by shRNA (200X). **G** The mRNA levels of transcription factors including *SP7*, *DLX5*, and *RUNX2* in hDPSCs infected by lentivirus containing empty vector (EV) or shOMD vector during osteogenic induction. Scale bar: 100 µm. Significance in difference between two groups were tested by Student’s *t*-test. Data were presented as mean ± SD.

The mRNA level of *OMD* significantly increased in the early period of osteogenesis, peaked on day 7 showing about 40-fold upregulation compared with that in GM, and maintained this level for about one week, before gradually decreased in the late period of osteogenesis (Fig. 1A). Though continuously increased level of OMD protein in cells cultured in GM was detected on day 1, 3, 7, more evident increase was shown in OM during the same period (Fig. 1C). In the late period of osteogenesis, though the intracellular level of OMD protein showed no significant differences between GM and OM (Fig. 1C), the supernatant level of OMD protein was still higher in OM than that in GM. (Fig. 1B). Next, the role of *OMD* gene on hDPSCs’ osteogenesis was investigated. Lower ALP activity was detected in OMD deficiency group (Fig. 1D, E). The knockdown efficiency of siRNAs was shown in Supplemental Fig. 1 and shRNA was indicated in a previous published paper^24^. Decreased mineralization was detected by Alizarin Red S (ARS) staining in shOMD after osteogenic induction for 21 days (Fig. 1F). The mRNA levels of osteogenic-related genes were also examined. In details, transcription factors such as *SP7* and *DLX5* presented different levels of significant down-regulation in shOMD and the late stage of induction witnessed the sharpest decline when compared to the EV group (Fig. 1G). Though the transcription of *RUNX2* upregulated transiently on day 1, it decreased visibly on day 14, 21 (Fig. 1G).

### **O**MD facilitates BMP2-induced osteogenesis in vitro and in vivo

**Fig. 2:**
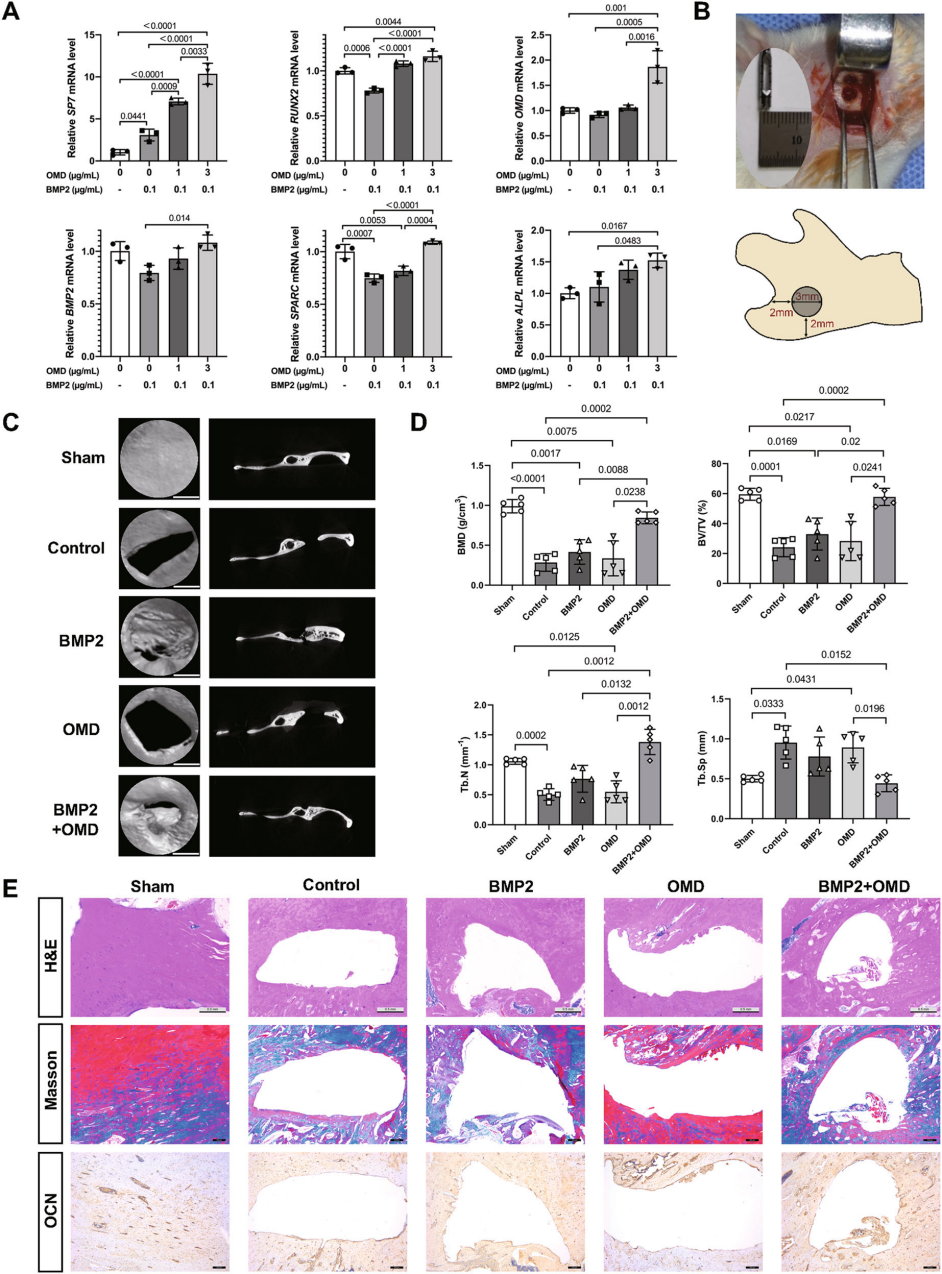
Effects of OMD on BMP2-induced osteogenic differentiation. **A** hDPSCs were cultured in FBS-free medium supplied with 0, 0.1 μg/mL BMP2 and 0, 1, 3 μg/mL OMD for 24 h. The mRNA levels of *SP7*, *RUNX2*, *OMD*, *BMP2*, *SPARC*, and *ALPL* in hDPSCs were examined by qPCR. Ordinary one-way ANOVA Tukey’s multiple comparison test was applied to evaluate statistical significance. **B** A full-thickness mandible bone defect of 3 mm in diameter was created with a dental trephine. **C** Newly formed bones were indicated by three-dimensional (3D) reconstructive images and two-dimensional (2D) images of mandibles. The images of the specimen whose value is closest to the group mean of bone volume/total volume (BV/TV) were presented as the “representative images”. Scale bar: 1 mm. **D** The bone mineral density (BMD), bone volume/total volume (BV/TV), trabecular bone number (Tb.N), and trabecular bone space (Tb.Sp) of the mandible defects were quantitated (*n* = 5). Brown-Forsythe or Welch’s ANOVA test with Games-Howell’s test for multiple comparisons. The sample power of BMD, BV/TV, Tb.N and Tb.Sp quantitated by software GPower 3.1 was 1.0000000, 1.0000000, 1.0000000 and 0.9873885 respectively. Data were presented as mean ± SD. **E** H&E staining (Scale bar: 0.5 mm), Masson’s trichrome staining (Scale bar: 200 μm) and immunohistochemical staining of Osteocalcin (OCN) (Scale bar: 200 μm) for bone tissue sections.

The extracellular effect of OMD was investigated with recombinant OMD protein. The cytotoxicity assay showed that the cell viability was not hampered within the concentration of 0–12 μg/mL of OMD (Supplemental Fig. 3). After serum starvation for 12 h, co-stimulation of hDPSCs with 0.1 μg/mL BMP2 and 0, 1, 3 μg/mL OMD for 24 h influenced osteogenic differentiation to various degrees. Specifically, *SP7*, a transcriptional activator essential for osteogenic differentiation, was prominently upregulated in an OMD concentration-dependent manner (Fig. 2A). Other osteogenic genes such as *RUNX2*, *OMD*, *BMP2*, *SPARC*, and *ALPL* showed upregulations with the addition of 3 μg/mL OMD when compared to other groups (Fig. 2A). As the addition of OMD enhanced the BMP2-induced osteogenesis in vitro, we assumed that OMD was able to optimize bone defect regeneration in vivo. To verify this assumption, four groups (Control; BMP2; OMD; BMP2 + OMD) of Puramatrix hydrogel were applied to the full-thickness mandible bone defect models, and sham operation group was applied as a positive control group. The representative 3D micro-CT reconstruction images and 2D images revealed the homogeneously dense bone formed in group BMP2 + OMD when compared to the Control, BMP2, or OMD group (Fig. 2C), which could also be revealed by H&E staining (Fig. 2E). The BMD, BV/TV and Tb.N in the BMP2 group were similar to those in control group or OMD group (Fig. 2D). However, when combined OMD with BMP2, the indexes including BMD, BV/TV and Tb.N were remarkably increased (Fig. 2D). Though Tb.Th showed no significant differences among the five groups (Supplemental Fig. 4), Tb.Sp of BMP2 + OMD group was significantly lower than that of Control group or OMD group (Fig. 2D). A characteristic bone feature with a red color inter-blended with blue was shown by Masson Trichrome staining. The BMP2 + OMD group has more mineralized bone (red) and less collagen-containing osteoid (blue) when compared with Control or BMP2 group (Fig. 2E). The defect area of OMD group appeared reddish in color, denoting mature bone formation (Fig. 2E). Immunohistochemical analysis confirmed an increased level of Osteocalcin (OCN), a protein secreted solely by osteoblasts, in the BMP2 + OMD group (Fig. 2E).

### **O**MD interacts with BMP2 and BMP cell surface receptors

**Fig. 3:**
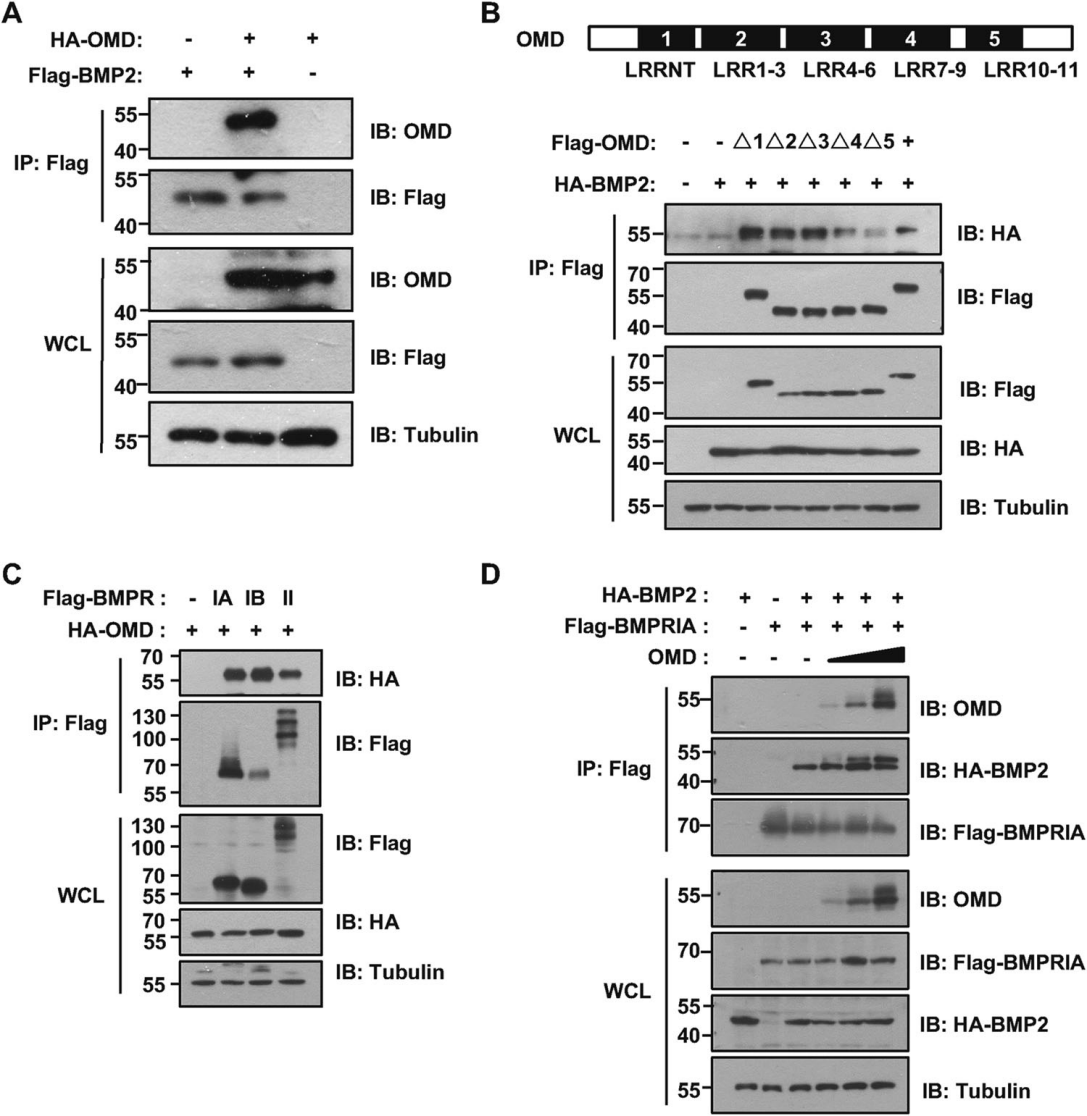
The interaction among OMD, BMP2, and BMP cell surface receptors. **A** The interaction of OMD and BMP2 was detected at exogenous level in 293 T cells by immunoprecipitation (IP). Immunoblot (IB) analysis of whole-cell lysates (WCL) with indicated antibodies was shown. **B** Immunoblot analysis of WCL and IP of 293T cells transfected with HA-BMP2 and different Flag-OMD truncates. **C** Flag-IP and IB analysis of 293T cells transfected with Flag-BMP receptors and HA-OMD plasmids. **D** 293T cells were transfected with OMD, Flag-BMPRIA and HA-BMP2 constructs as indicated and cell lysates were harvested for IP and IB analysis.

The relationship between OMD and BMP2 was in-depth analyzed because of their synergistic effect on osteogenesis. The strong binding of OMD and BMP2 was detected by Co-IP of ectopically expressing OMD and BMP2 in 293T cells (Fig. 3A). In an effort to identify whether the LRRs were responsible for the interaction of OMD with BMP2, a series of OMD deletion constructs were made (shown schematically in Fig. 3B). One of the five mutants, Δ5, which was lack of the tenth and eleventh LRRs, had significantly impaired interaction with BMP2 protein when compared with full-length OMD and other deletion constructs (Fig. 3B). To investigate whether OMD’s binding with BMP2 could disturb the interaction of BMP2 with its cell surface receptors, we performed the Co-IP assay using type I and type II BMP receptors (Fig. 3C). The results showed that OMD could bind to type II BMP receptor. Meanwhile, complexes of OMD with type IA or type IB BMP receptor were also immunoprecipitated. As BMP2 exhibits a higher affinity to the type I than the type II receptor^27^, the BMPRIA was selected to demonstrate the effect of OMD on BMP2-receptor interaction. It is worth noting that OMD did not interfere or decrease the interaction between BMP2 and BMPRIA (Fig. 3D). In addition, OMD promoted the interaction between BMPRIA and BMP2 in a concentration-dependent manner, indicating that a complex comprising OMD, BMP2 and its receptor could form and the presence of OMD did not hinder the interaction between BMP2 and BMPRIA.

### **O**MD induces osteogenic differentiation through BMP/SMAD pathway

**Fig. 4:**
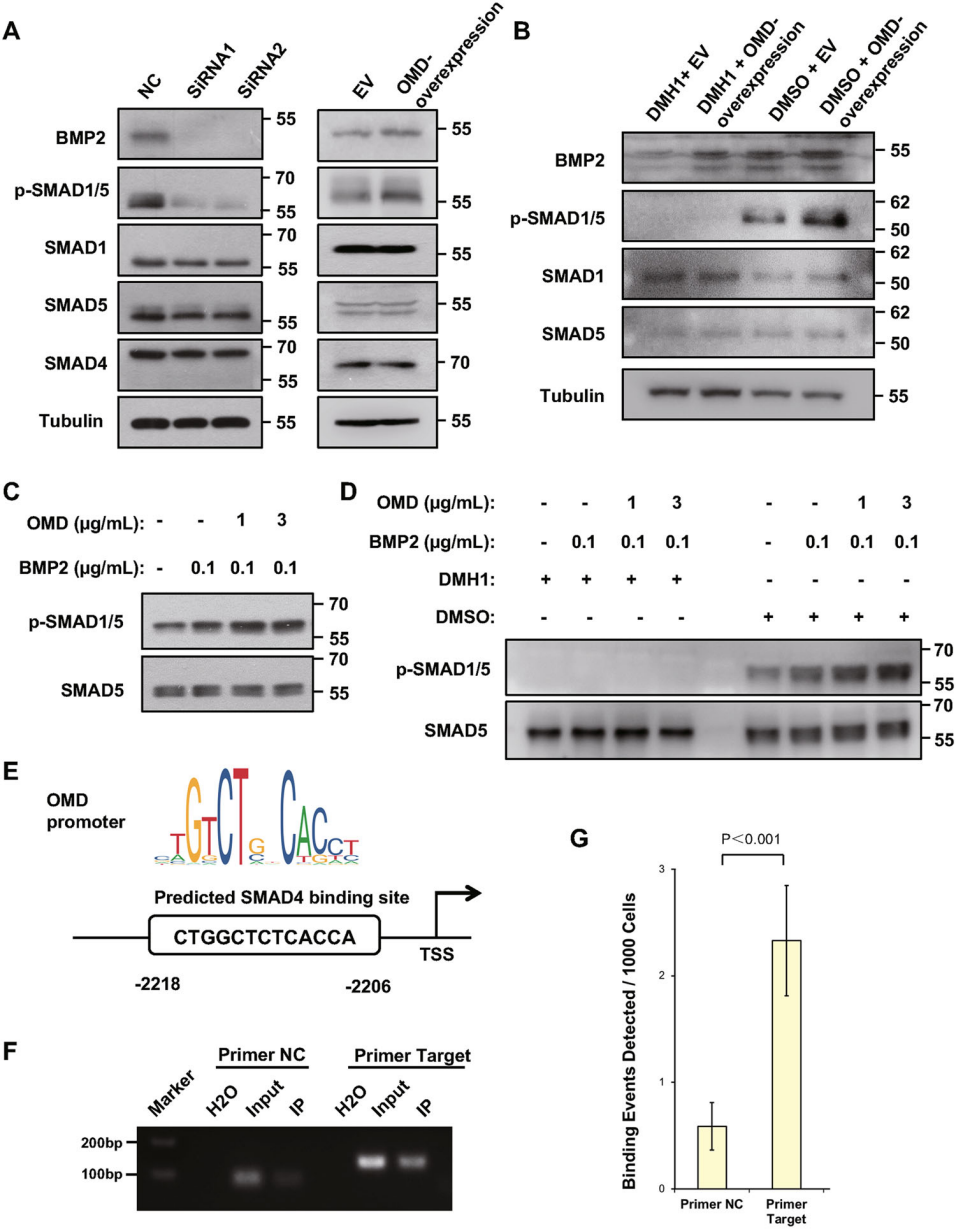
Effects of OMD on the BMP2-SMAD signaling pathway in hDPSCs. **A** The levels of BMP2, phosphorylated SMAD1/5, and total SMAD1/4/5 were examined after OMD was silenced or overexpressed. **B** After treatment with 20 μM DMH1 to block BMP type I receptor or equal volume DMSO as controls for 24 h, the activation of BMP2/SMAD pathway was checked in hDPSCs overexpressing OMD or empty vector (EV). **C** hDPSCs were cultured in 5% FBS medium supplied with different concentrations of BMP2 and OMD as shown for 1 h, and then the phosphorylated SMAD1/5 and total SMAD5 were examined. **D** hDPSCs were cultured in 5% FBS medium supplied with designated concentrations of BMP2 and OMD as well as 20 μM DMH1 or equal volume DMSO for 1 h. The protein levels of phosphorylated SMAD1/5 and total SMAD5 were examined in hDPSCs via immunoblots. **E** Schematic of the significantly enriched motif in SMAD4 binding region predicted by FIMO and JASPAR program. **F** ChIP assays assessing SMAD4 binding to the *OMD* promoter in hDPSCs by agarose gel electrophoresis. **G** SMAD4-bound DNA was quantified by qPCR and expressed as binding events detected per 1000 cells. Enrichment relative to negative control primers is indicated.

After demonstrating that OMD could enhance BMP2-induced osteogenic differentiation in vitro and in vivo, we investigated the effect of OMD on BMP2 downstream signaling. OMD knockdown by siRNA decreased BMP2 expression and subsequently impaired SMAD1/5 phosphorylation in hDPSCs, while lentivirus-mediated OMD overexpression reversed such effect (Fig. 4A). The overexpression efficiency of OMD was confirmed by immunoblot (Supplemental Fig. 2). DMH1, which specifically targets the intracellular kinase domain of BMP type I receptors^28^, was used to block BMP signaling. The results indicated that the activation of SMAD signaling induced by OMD overexpression could be interrupted by BMP receptor blocker (Fig. 4B). The phosphorylation level of SMAD1/5 was higher in the hDPSCs treated with the two recombinant biofactors than in the cells treated with BMP2 alone, and was positively relevant to the dose of OMD with the existence of BMP2 (Fig. 4C). Similarly, DMH1 could also abolish the SMAD1/5 phosphorylation which was triggered by a combination of OMD and BMP2 proteins (Fig. 4D). To explore the genomic binding positions of SMADs in the promoter region of *OMD*, a motif scanner program FIMO was used to determine the putative binding sites of motif predicted by JASPAR CORE database with default parameters^29,30^. The results showed that there was 1 motif occurrence of SMAD4 with a *p*-value less than 0.0001 (*p*-value 1.04e − 5, *q*-value 0.0625), which was also the highest ranking sequence predicted by JASPAR database (Fig. 4E). It located within 3 kb upstream of the transcription start site (TSS) of *OMD* gene, and its binding affinity was verified by ChIP-qPCR assay (Fig. 4F, G).

## Discussion

We have previously shown that OMD-silenced hDPSCs exhibited lower osteoblastic differentiation ability compared with controls^24^. Here, we demonstrated that the expression pattern of OMD was robustly correlated with osteogenic phase. The positive function of OMD in osteogenesis was further verified by evaluating molecular markers of osteoblastic lineage. A deeper investigation of the mechanisms showed that OMD could activate canonical BMP/SMAD pathway, and enhance BMP2-induced SMAD signaling, osteogenic gene expression as well as bone formation. This activation could be completely inhibited by BMPRI inhibitor, confirming the function of OMD in mediating BMP/SMAD signal transduction. The potential interaction among OMD, BMP2 and its membrane receptors may explain the aforementioned effects.

Majority of ECM components are the proteoglycans, among which the SLRPs are the largest family^31^. SLRPs are capable of clustering different types of receptors and affecting downstream intracellular phosphorylation events, including those driven by TGF-beta/BMP superfamily members, epidermal growth factor receptors, insulin like growth factor I receptors, Met receptor and Toll-like receptors^32,33^. In addition to cellular proliferation^33^, inflammation^34^ and innate immunity^35^, it has also been well-established that specific SLRPs are functionally involved in bone osteogenesis and homeostasis^11^. Unlike the well-studied SLRP members such as decorin and biglycan, the entirety of OMD’s biological functions remains elusive. In light of structural similarity and multifunction of SLRP members, therefore, it is hypothesized OMD may share some typical features of its group.

The principle role of OMD in skeletal development is corroborated by the observation that abundant expression of OMD has been detected within mature osteoblasts derived from MC3T3E1 cell lines or primary osteoblasts^22,36,37^. It has been reported that OMD may be secreted into the pericellular space of odontoblasts and osteoblasts and serve as a “fossilized” protein after biomineralization^15,18^. In current study, the expression of OMD in hDPSCs, a type of multipotent cells capable of osteoblastic differentiation^38^, significantly increased when cultured in osteogenic medium. In the late period of osteogenesis, OMD protein may be secreted into the extracellular environment, thus taking a part in matrix assembly or cellular modulation. The knockdown of *OMD* in hDPSCs could attenuate the ALP activity and calcium deposits, which coincides well with previous studies^37^. Unsurprisingly, it could also influence the molecular markers of osteoblastic lineage. RUNX2, SP7 and DLX5 are mandatory transcriptional factors regulating the osteogenic process^39,40^. The overall transcription levels of *SP7* and *DLX5* were significantly declined following knockdown of *OMD* during osteogenesis. Intriguingly, *RUNX2* was transiently upregulated in the early period, indicating that some antagonizing pathway of short duration may be aroused.

BMPs are initiators of a biological cascade that involves osteogenic signaling events and that culminates in the production of functional bone tissue^41^. BMP2 is a potent stimulator of osteoblastogenesis. It is known that *RUNX2* and *SP7* are well-established BMP2-target genes and *DLX5* is required for SP7 expression under BMP signaling^42,43^. Owing to the synergistic effect between these transcription factors and BMP2, it is, therefore, feasible to assume that OMD may be implicated in the BMP/SMAD signaling. It was found that the forced expression of OMD could increase the level of BMP2 and elicit activation of the canonical BMP pathway effectors SMAD1 and 5. Conversely, suppression of OMD down-regulated BMP2 and then prevented the intracellular signal transduction. Previous studies show that BMP2 is capable of inducing the expression of OMD^22,23^. To further elucidate the underlying molecular mechanism, the ChIP assay was performed and showed that SMAD4 endogenously associated with region 2.2 kb upstream of TSS in *OMD* promoter. Thus, these data suggest a possible feed-forward cycle between OMD and BMP2, which may drive the osteogenic response forward.

Recombinant BMPs have been exploited as osteoinductive agents of bone healing in pre-clinical models or clinical settings^44^. Low dosages of BMP2 exhibit inferior osteogenic ability or even induce the commitment of stem cells into adipocytes^45^. In this work, 100 ng/mL BMP2 was able to prominently stimulate *SP7* expression in hDPSCs albeit low expression of *RUNX2*. This is in consent with previous reports that BMP2-induced SP7 expression can occur independently of RUNX2^42,46^. With the coexistence of BMP2, OMD could enhance the expression of osteogenic genes and the phosphorylation of SMAD1/5 in a dose-dependent manner. This effect could be recapitulated in vivo with a rat mandibular bone defect model, which showed that exogenous addition of OMD could accelerate BMP2-induced bone healing. Mineralized bone forms when collagen-containing osteoid integrates hydroxyapatite crystals^47^. The Masson’s trichrome staining potentially indicated that OMD accelerated mineral accrual when bone was remodeled. Of note, the SP7 transcription factor showed significant correlation with OMD and this implied that OMD could activate signaling targeted at SP7, which may underlie the accelerated bone formation. Another possible mechanism behind this effect was that OMD may sequester BMP2 in the ECM for sustainable releasing.

The relationship between OMD and BMP2 was further investigated because of their synergistic effect. Our study indicated that OMD had a high affinity to BMP2. The structure of OMD contains 11 LRR motifs, which are the characterized feature of the core protein^14^. It has been documented that LRRs are considered to be sites of protein–protein interactions^48^. In the solenoid structures of LRRs, the concave surface is often used for protein or ligand binding and the mutations frequently affect the protein/ligand affinity^49^. In this regard, the significance of different LRR sequences of OMD was verified in the interaction. As noted in previous studies, OMD binding to type I collagen is driven by weak electrostatic forces involving the residues Glu284 (located in LRR9) and Glu303 (located in LRR10)^16^. Our results showed that the tenth and eleventh of LRRs were essential parts for the binding between OMD and BMP2, and were presumed to be the potential effective domain facilitating BMP2-induced signal transduction. Asporin, the third member of the type I SLRPs, possesses an opposite feature of inactivation of the BMP2 signaling pathway via its LRR5 motif, exemplifying diverse effects of LRRs^50^.

**Fig. 5:**
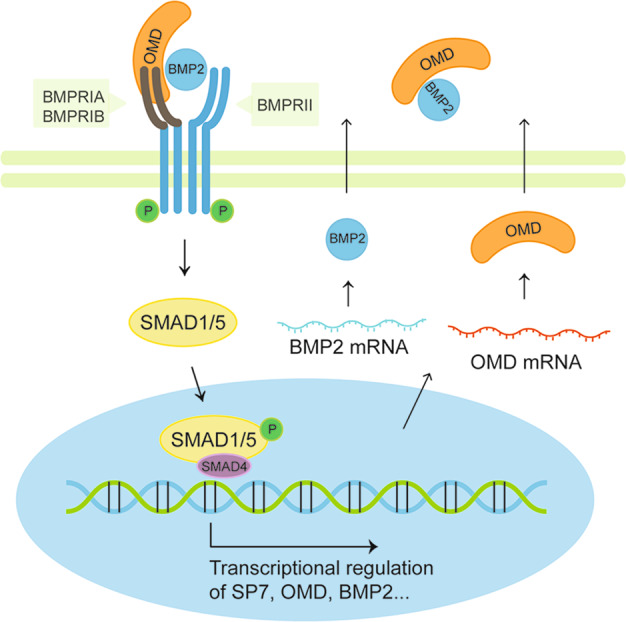
Schematic representation of the mechanism by which OMD mediates osteogenesis and acts as a coordinator of BMP2/SMAD pathway. During the process of osteogenic differentiation, the expression of OMD is elevated in hDPSCs. The mature OMD protein interacts with BMP2 cytokine via its C-terminal leucine-rich repeats and clusters with BMP receptors, which may facilitate BMP2 to anchor its cell surface receptors and further ignite intracellular signal transmission. The nuclear translocation of phosphorylated SMAD1/5 activates the transcription of osteogenic-associated genes such as *SP7*, *OMD* and *BMP2*, and consequently exert biological effects.

BMP2 transduces signals by complexing with transmembrane serine-threonine kinase receptors^51^. The receptors are classified into two subgroups termed type I and type II. Type I receptor can be phosphorylated as a downstream component of type II receptors, and determine the specificity of the intracellular signals in BMP signaling^52^. The specific interaction of OMD with the two types of receptors were investigated in this study, which indicated that OMD had binding affinities for both types of BMP receptors. Furthermore, it seemed that the BMPRIB exhibited higher affinity with OMD than other receptor subsets in the interaction analysis. It has been identified that BMP2 binds BMPRIA with at least 50- to 60- fold higher affinity than BMPRII^53^. Consequently, whether the binding effect of OMD with BMPRIA would interfere the interaction between BMP2 and BMPRIA was explored. It is noteworthy that OMD did not interfere or hide the sites of interaction between BMP2 and BMPRIA. In other words, the complex formed by OMD, BMP2 and its receptors presumably promote BMP2 to anchor its cell surface receptors. The subsequent signal transduction could be abolished by BMP type I receptor inhibitor, which further support the osteogenic role of OMD via BMP2/SMAD pathway. However, much remains to be discovered about the exact function of OMD in the detailed structural assembly and conformational changes of BMP2/BMPRI/BMPRII ternary complex. The possible regulatory mechanisms of OMD are summarized in Fig. 5.

In conclusion, the present study provides evidence for what is, to our knowledge, a novel role of the matrix component OMD as a positive modulator of osteogenesis and a coordinator of BMP2 signaling. The combination of BMP2 and OMD manifest enhanced biological activity. The C-terminal leucine-rich repeats in OMD are presumed to be the main domain of interaction with BMP2. OMD may facilitate BMP2 to anchor its cell surface receptors and further ignites intracellular signal transmission. The location and function of OMD outside the cell makes it easier to intervene and exogenous addition of OMD may potentially be a new approach for bone regeneration strategy but more in-depth insight into OMD’s biology is warranted with regard to risk management and treatment efficacy.

## Supplementary information

Supplemental Figure 1 to 4

Supplemental Table 1. Primers used for qRT-PCR and ChIP

Supplemental Table 2. SiRNA sequences

Supplemental Table 3. Antibodies

Supplemental Table 4. Biological modulators
