# Correction: Osteomodulin positively regulates osteogenesis through interaction with BMP2

**DOI:** 10.1038/s41419-025-08120-y

**Published:** 2026-01-13

**Authors:** Wenzhen Lin, Xiaohan Zhu, Li Gao, Mengying Mao, Daming Gao, Zhengwei Huang

**Affiliations:** 1 Department of Endodontics, Shanghai Ninth People’s Hospital, College of Stomatology, Shanghai Jiao Tong University School of Medicine, Shanghai, China; 2 National Clinical Research Center for Oral Diseases, Shanghai, China; 3 Shanghai Key Laboratory of Stomatology & Shanghai Research Institute of Stomatology, Shanghai, China; 4 Department of Endodontics, Shanghai Stomatological Hospital, Fudan University, Shanghai, China; 5 Oral Biomedical Engineering Laboratory, Shanghai Stomatological Hospital, Fudan University, Shanghai, China; 6 State Key Laboratory of Cell Biology, CAS Key Laboratory of Systems Biology, CAS Center for Excellence in Molecular Cell Science, Shanghai Institute of Biochemistry and Cell Biology, Chinese Academy of Sciences, Shanghai, China; 7 University of Chinese Academy of Sciences, Beijing, China

Correction to: *Cell Death and Disease* 10.1038/s41419-021-03404-5, published online 01 February 2021

During a thorough review of our published work, we identified an image error in Figure 1E, which presents both overview and magnified images of ALP staining for siRNA1 and siRNA2 (both targeting the OMD gene for knockdown). While the results demonstrated that OMD knockdown by either siRNA reduced ALP activity, as clearly illustrated in the overview images, we regrettably discovered that the magnified ALP staining image labeled as siRNA2 was inadvertently imported from siRNA1 due to insufficient image annotation during figure assembly.

This was an unintentional oversight, and we sincerely apologize for this error. We have the original, uncropped overview and magnified ALP staining images for both siRNA1 and siRNA2 to support the validity of our experimental results. This correction does not affect the data interpretation or the study’s conclusions.

Incorrect Figure 1E

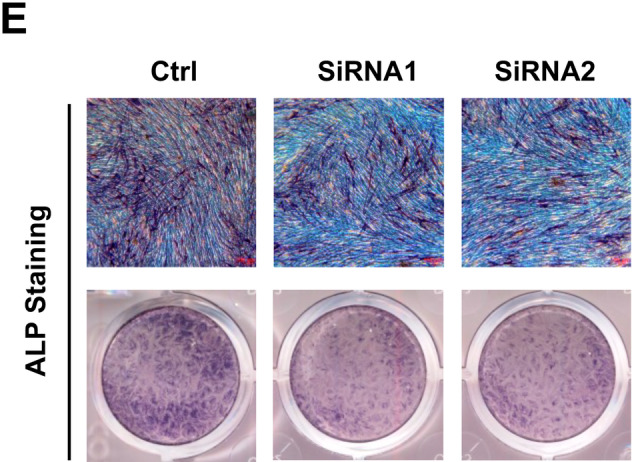



Corrected Figure 1E

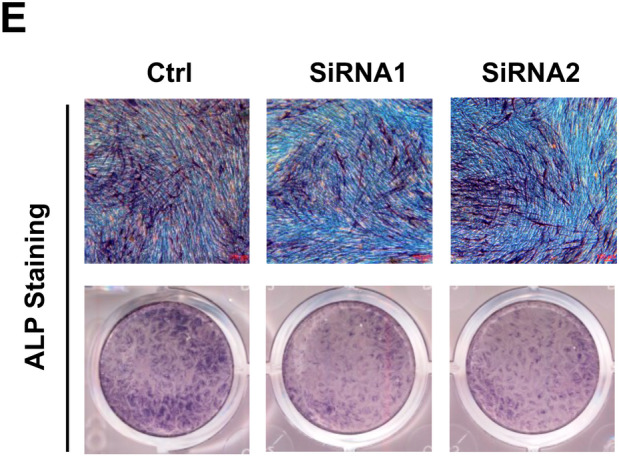



The original article has been corrected.

## Supplementary information

Amended data

Original data

