# Supplementary material for: Osteomodulin positively regulates osteogenesis through interaction with BMP2
Source: Cell Death Dis. 2021 Feb 1;12(2):147. doi: 10.1038/s41419-021-03404-5 (PMC7862363; doi:10.1038/s41419-021-03404-5)
Supplement: Supplementary file 1 — Supplemental Figure 1 to 4 [file 41419_2021_3404_MOESM1_ESM.docx]

**
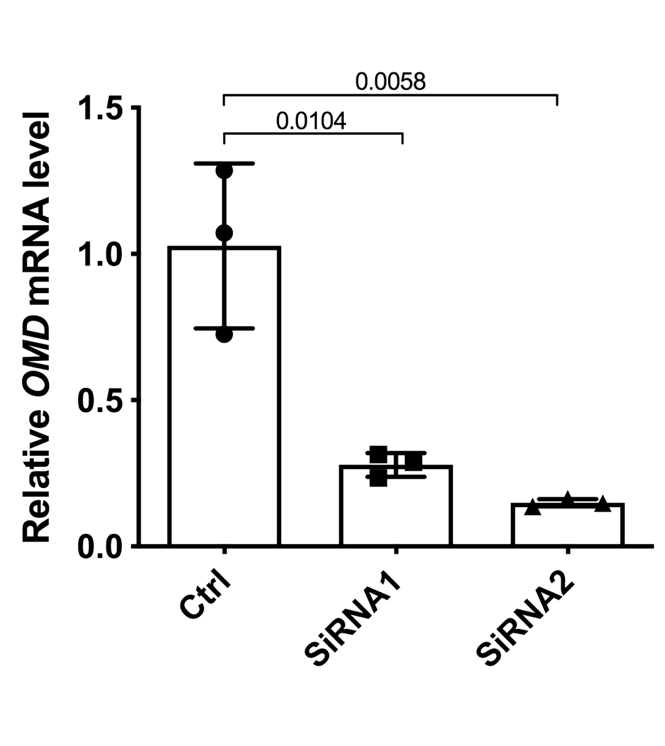
**

**Supplemental Figure 1.** The efficacy of the siRNA target *OMD* gene was quantified by qPCR. Significance in difference between the control group and siRNA group were tested by Student’s *t*-test.

**
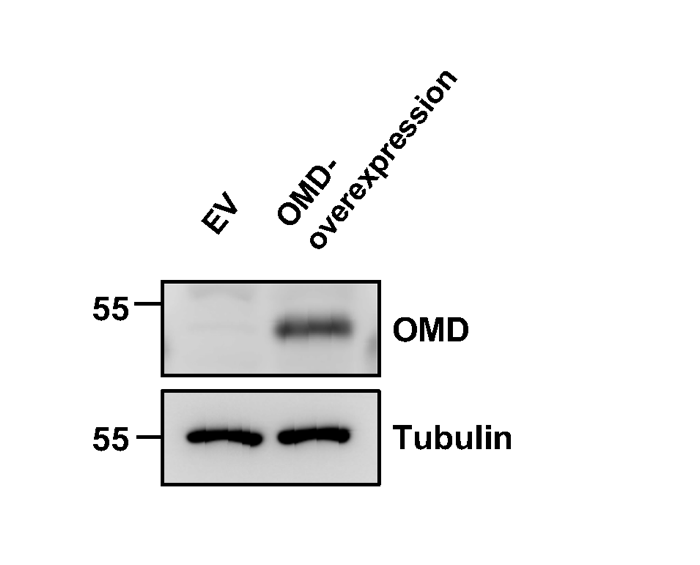
**

**Supplemental Figure 2.** OMD protein level in EV group and OMD overexpression group of hDPSCs.

**
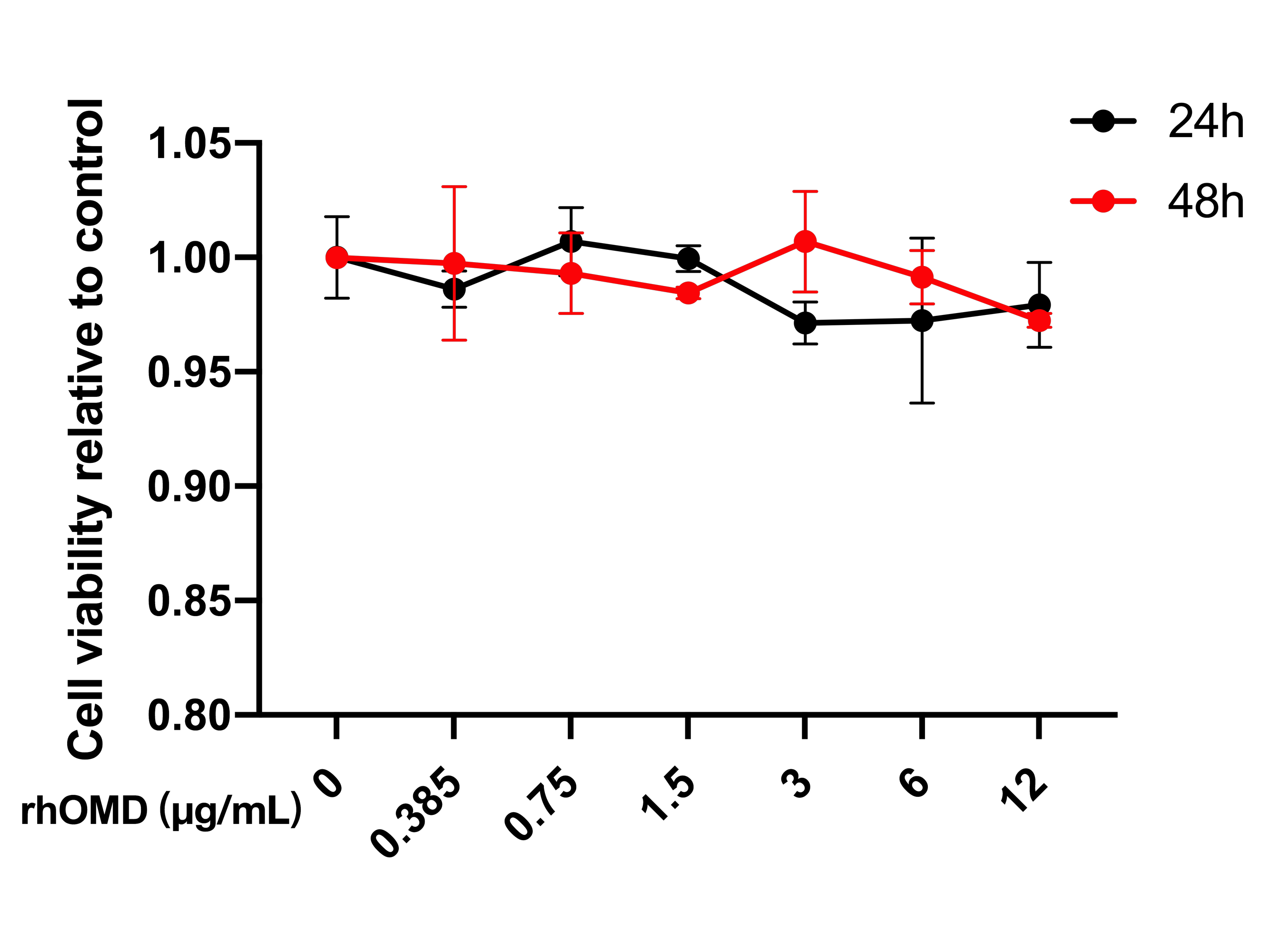
**

**Supplemental Figure 3.** The cytotoxicity of rhOMD on hDPSCs.


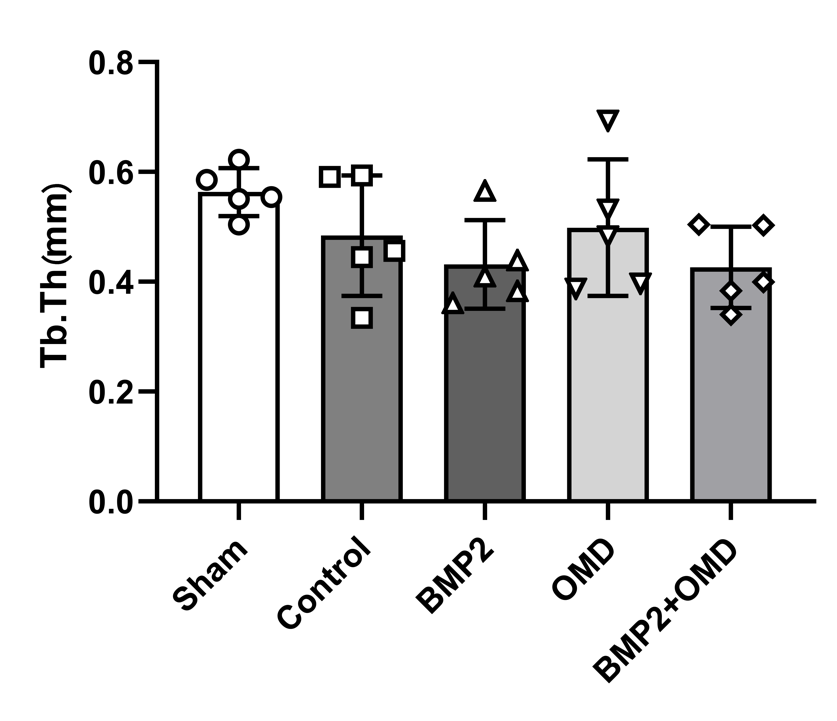


**Supplemental Figure 4.** The trabecular thickness (Tb.Th) index of the mandible defects. Tb.Th showed no significant differences among the five groups. One-way ANOVA with Tukey’s post hoc test based on normal distributions and equal variance assumptions tests was used for multiple comparisons.
