# Supplementary material for: Osteomodulin positively regulates osteogenesis through interaction with BMP2
Source: Cell Death Dis. 2021 Feb 1;12(2):147. doi: 10.1038/s41419-021-03404-5 (PMC7862363; doi:10.1038/s41419-021-03404-5)
Supplement: Supplementary file 2 — Supplemental Table 1. Primers used for qRT-PCR and ChIP [file 41419_2021_3404_MOESM2_ESM.docx]

**Supplemental Table 1. Primers used for qRT-PCR and ChIP**

| **Gene ID** | **Target/**  **Primer Name** | **Primer sequence** | **Amplicon**  **size (bp)** | **Source** | **Assay** |
| --- | --- | --- | --- | --- | --- |
| 60 | *ACTB* | (F) TGGCACCCAGCACAATGAA | 186 | ^1^ | qPCR |
|  |  | (R) CTAAGTCATAGTCCGCCTAGAAGCA |  |  |  |
| 249 | *ALPL* | (F) CCTTGTAGCCAGGCCCATTG | 137 | ^2^ | qPCR |
|  |  | (R) GGACCATTCCCACGTCTTCAC |  |  |  |
| 650 | *BMP2* | (F) GTCCTGAGCGAGTTCGAGTT | 115 | ^3^ | qPCR |
|  |  | (R) AGTGCCTGCGATACAGGTCT |  |  |  |
| 1749 | *DLX5* | (F) TTCCAAGCTCCGTTCCAGAC | 86 | Primer | qPCR |
|  |  | (R) GAATCGGTAGCTGAAGACTCG |  | Bank |  |
| 4958 | *OMD* | (F) AGGCTGTGTCAGTGAATGCT | 107 | ^4^ | qPCR |
|  |  | (R) GTTGCTGAATGTGCATCGGA |  |  |  |
| 860 | *RUNX2* | (F) CACTGGCGCTGCAACAAGA | 127 | ^2^ | qPCR |
|  |  | (R) CATTCCGGAGCTCAGCAGAATAA |  |  |  |
| 6678 | *SPARC* | (F) TTCCCTGTACACTGGCAGTTC | 109 | ^5^ | qPCR |
|  |  | (R) AATGCTCCATGGGGATGA |  |  |  |
| 121340 | *SP7* | (F) CATTCTGGGCTTGGGTATCT | 93 | ^6^ | qPCR |
|  |  | (R) GGCCTGAGATGAGAGTTTGT |  |  |  |
| 4958 | Primer Target | (F)TGTCACCACCTCACCTCGCTTA | 130 | Primer | ChIP |
|  |  | (R)CAGTTCAGGCACCGCTGTATGT |  | premier |  |

Reference for primers

1. Li L, Liu X, Sanders KL, et al. TLR8-Mediated Metabolic Control of Human Treg Function: A Mechanistic Target for Cancer Immunotherapy. *Cell Metab.* 2019;29(1):103-123 e105.

2. Ma QL, Zhao LZ, Liu RR, et al. Improved implant osseointegration of a nanostructured titanium surface via mediation of macrophage polarization. *Biomaterials.* 2014;35(37):9853-9867.

3. Bai Q, Yan H, Sheng Y, et al. Long-term acetaminophen treatment induced liver fibrosis in mice and the involvement of Egr-1. *Toxicology.* 2017;382:47-58.

4. Lin W, Gao L, Jiang W, et al. The role of osteomodulin on osteo/odontogenic differentiation in human dental pulp stem cells. *BMC oral health.* 2019;19(1):22.

5. Brun P, Ghezzo F, Roso M, et al. Electrospun scaffolds of self-assembling peptides with poly(ethylene oxide) for bone tissue engineering. *Acta Biomater.* 2011;7(6):2526-2532.

6. Kong X, Liu Y, Ye R, et al. GSK3beta is a checkpoint for TNF-alpha-mediated impaired osteogenic differentiation of mesenchymal stem cells in inflammatory microenvironments. *Biochim Biophys Acta.* 2013;1830(11):5119-5129.
