# Supplementary material for: Osteomodulin positively regulates osteogenesis through interaction with BMP2
Source: Cell Death Dis. 2021 Feb 1;12(2):147. doi: 10.1038/s41419-021-03404-5 (PMC7862363; doi:10.1038/s41419-021-03404-5)
Supplement: Supplementary file 3 — Supplemental Table 2. SiRNA sequences [file 41419_2021_3404_MOESM3_ESM.docx]

**Supplemental Table 2. SiRNA sequences**

| Name | Direction | Base sequences |
| --- | --- | --- |
| OMD-siRNA1 | Forward (5'-3') | GCCAAUAUGAAACUUAUCAdTdT |
|  | Reverse (5'-3') | UGAUAAGUUUCAUAUUGGCdTdT |
| OMD-siRNA2 | Forward (5'-3') | CUACAAGACAUCCCAUAUAdTdT |
|  | Reverse (5'-3') | UAUAUGGGAUGUCUUGUAGdTdT |
| Ctrl-siRNA | Forward (5'-3') | UUCUCCGAACGUGUCACGUdTdT |
|  | Reverse (5'-3') | ACGUGACACGUUCGGAGAAdTdT |
