# Supplementary material for: Osteomodulin positively regulates osteogenesis through interaction with BMP2
Source: Cell Death Dis. 2021 Feb 1;12(2):147. doi: 10.1038/s41419-021-03404-5 (PMC7862363; doi:10.1038/s41419-021-03404-5)
Supplement: Supplementary file 4 — Supplemental Table 3. Antibodies [file 41419_2021_3404_MOESM4_ESM.docx]

**Supplemental Table 3. Antibodies**

| **Epitope/Antigen or Product name** | **Source and Catalog # or RRID** | **Host species** | **Application (WB, IP, F, IHC, ICC, CHIP, N)** | **Dilution** | **Application specific details (e.g. antigen retrieval, blocking, incubation)** |
| --- | --- | --- | --- | --- | --- |
| *OMD* | *Abclonal, #A12256* | *Rabbit* | *WB* | *1:1000* | *5% BSA, overnight, 4℃* |
| *α Tubulin* | *Santa Cruz Biotechnology, #SC-23948* | *Mouse* | *WB* | *1:1000* | *5% BSA, overnight, 4℃* |
| *BMP2* | *Abcam, #ab14933* | *Rabbit* | *WB* | *1:500* | *5% BSA, overnight, 4℃* |
| *p-SMAD1/5* | *CST, #9516T* | *Rabbit* | *WB* | *1:1000* | *5% BSA, overnight, 4℃* |
| *SMAD1* | *CST, #6944T* | *Rabbit* | *WB* | *1:1000* | *5% BSA, overnight, 4℃* |
| *SMAD5* | *CST, #12534T* | *Rabbit* | *WB* | *1:1000* | *5% BSA, overnight, 4℃* |
| *SMAD4* | *CST, #38454T* | *Rabbit* | *WB* | *1:1000* | *5% BSA, overnight, 4℃* |
| *SMAD4* | *CST, #38454T* | *Rabbit* | *ChIP* | *1:100* | *Overnight, 4℃* |
| *HA-Tag* | *Santa Cruz Biotechnology, #SC-805* | *Rabbit* | *Co-IP* | *1:1000* | *5% BSA, overnight, 4℃* |
| *Flag* | *Proteintech, #* *20543-1-AP* | *Rabbit* | *Co-IP* | *1:1000* | *5% BSA, overnight, 4℃* |
| *HA-Tag* | *Santa Cruz Biotechnology, #* *SC-7392* | *Mouse* | *Co-IP* | *1:1000* | *5% BSA, overnight, 4℃* |
| *Flag-Tag* | *Sigma-Aldrich, #F3165* | *Mouse* | *Co-IP* | *1:1000* | *5% BSA, overnight, 4℃* |
| *OCN* | *Santa Cruz Biotechnology, #SC-30045* | *Mouse* | *IHC* | *1:100* | *5% Serum, overnight, 4℃* |
