# Supplementary material for: Osteomodulin positively regulates osteogenesis through interaction with BMP2
Source: Cell Death Dis. 2021 Feb 1;12(2):147. doi: 10.1038/s41419-021-03404-5 (PMC7862363; doi:10.1038/s41419-021-03404-5)
Supplement: Supplementary file 5 — Supplemental Table 4. Biological modulators [file 41419_2021_3404_MOESM5_ESM.docx]

**Supplemental Table 4. Biological modulators**

| **Modulator (protein, small molecule)** | **Source,** **Catalog # or RRID** | **Solvent/Vehicle** | **Concentration(s)** |
| --- | --- | --- | --- |
| *rhBMP2* | *PeproTech, #120-02* | *0.1% BSA* | *0.1 μg/mL* |
| *rhOMD* | *Sino Biological, #10825-H08H* | *0.1% BSA* | *0-3 μg/mL* |
| *DMH1* | *Selleck, #S7146* | *DMSO* | *20 μM* |
