# Supplementary figures and images for: Correction: Osteomodulin positively regulates osteogenesis through interaction with BMP2
Source: Cell Death Dis. 2026 Jan 13;17(1):29. doi: 10.1038/s41419-025-08120-y (PMC12800333; doi:10.1038/s41419-025-08120-y)

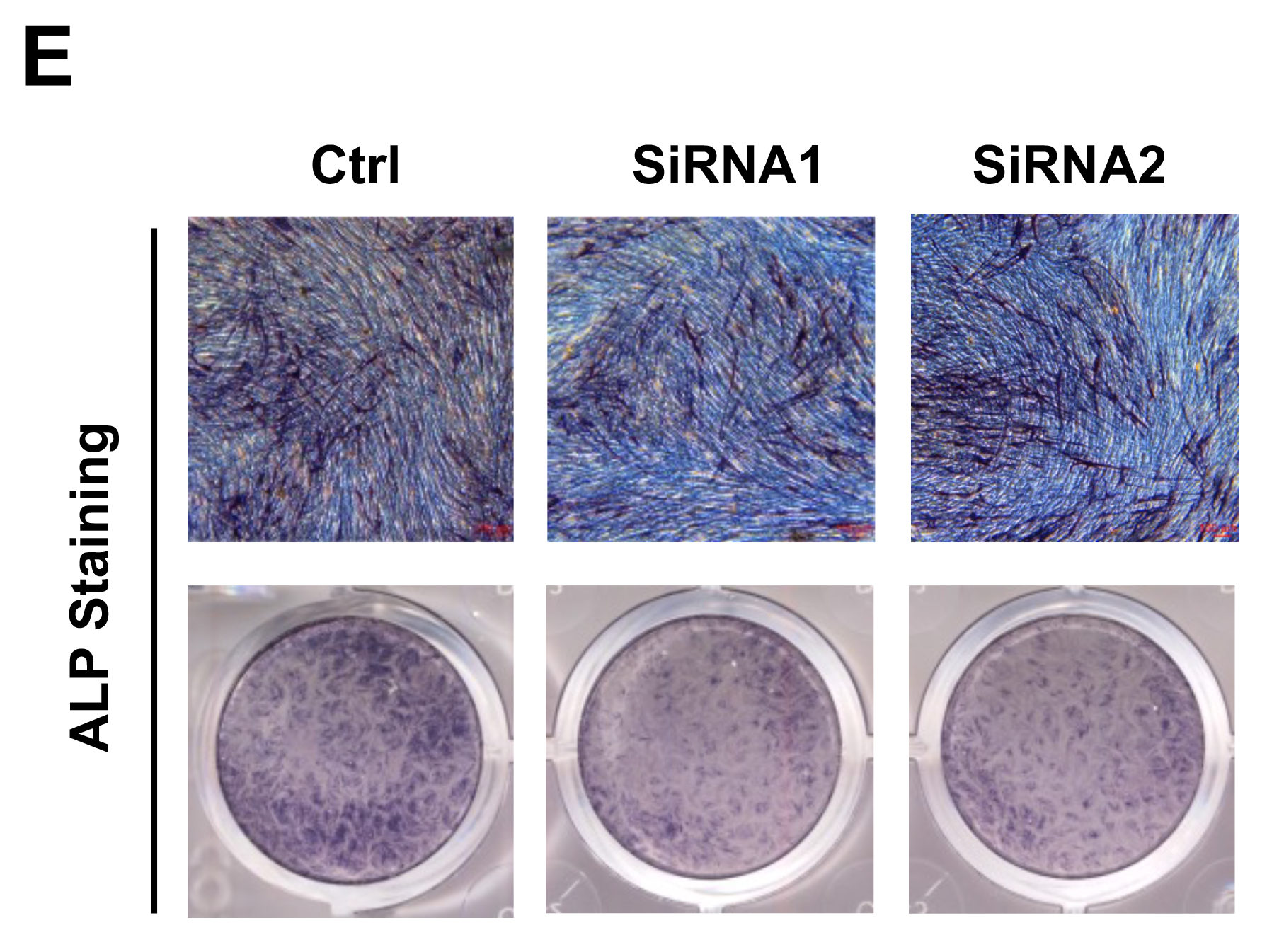

Supplement: Supplementary file 1 — Amended data [file 41419_2025_8120_MOESM1_ESM.tif]

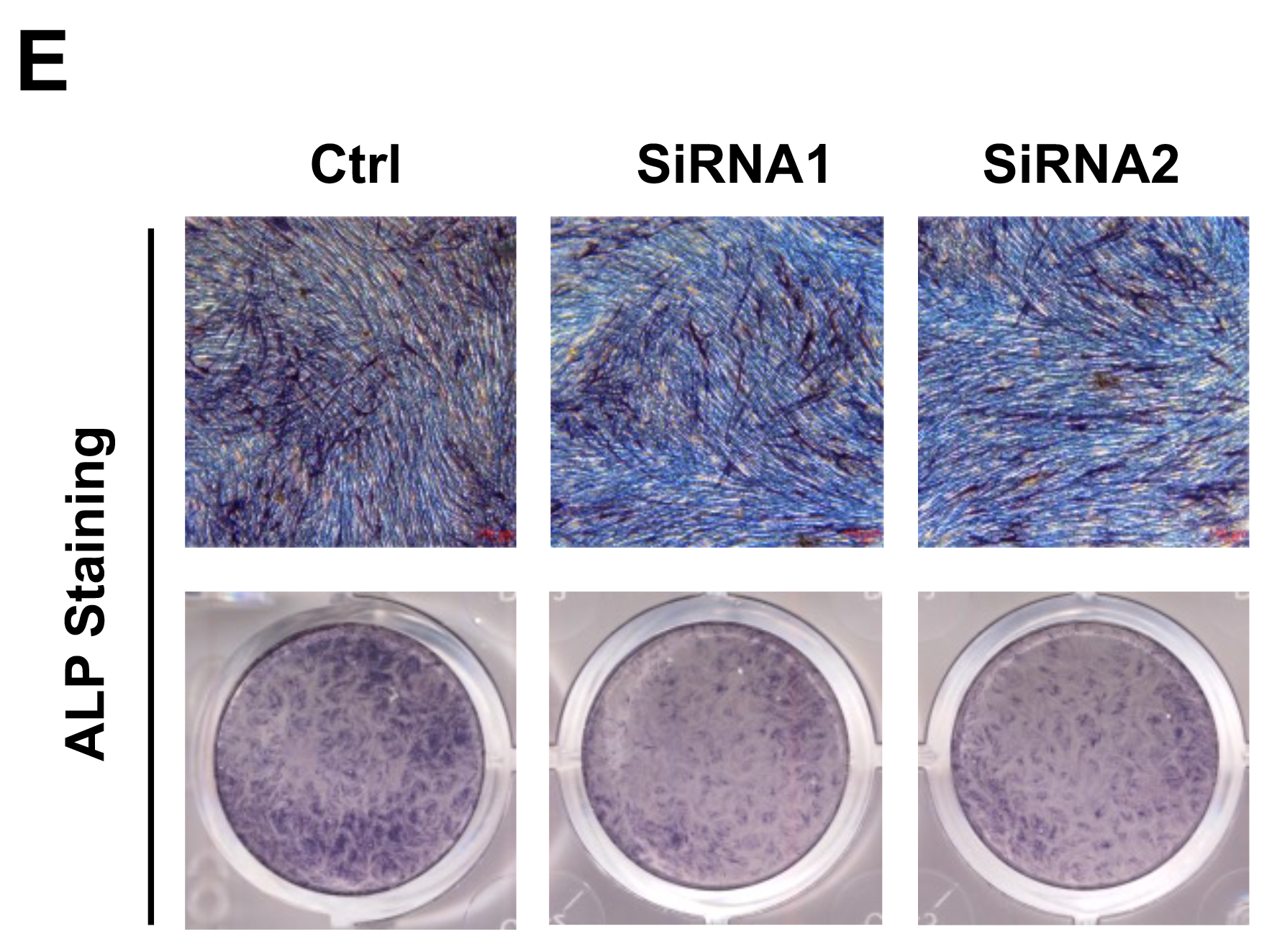

Supplement: Supplementary file 2 — Original data [file 41419_2025_8120_MOESM2_ESM.tif]
